# Italian Validation of the 12-Item Version of the Burnout Assessment Tool (BAT-12)

**DOI:** 10.3390/ijerph19148562

**Published:** 2022-07-13

**Authors:** Greta Mazzetti, Chiara Consiglio, Ferdinando Paolo Santarpia, Laura Borgogni, Dina Guglielmi, Wilmar B. Schaufeli

**Affiliations:** 1 Department of Education Studies “G. M. Bertin”, Alma Mater Studiorum—Università di Bologna, Via Filippo Re, 6, 40126 Bologna, Italy; dina.guglielmi@unibo.it; 2 Department of Psychology, Faculty of Medicine & Psychology, Sapienza University of Rome, Via dei Marsi, 78, 00185 Rome, Italy; chiara.consiglio@uniroma1.it (C.C.); ferdinandopaolo.santarpia@uniroma1.it (F.P.S.); laura.borgogni@uniroma1.it (L.B.); 3 Research Unit Occupational & Organizational Psychology and Professional Learning, KU Leuven, Dekenstraat 2, 3000 Leuven, Belgium; wilmar.schaufeli@kuleuven.be or; 4 Department of Psychology, Utrecht University, P.O. Box 80.140, 3508 TC Utrecht, The Netherlands

**Keywords:** burnout, BAT, burnout assessment tool, psychometric properties, validation, JD-R model, health impairment process, exhaustion, COVID-19

## Abstract

The Burnout Assessment Tool (BAT) has shown satisfactory validity evidence in several countries, with the 23-item version of the instrument reporting adequate psychometric properties also in the Italian context. This paper is aimed to present results from the Italian validation of the 12-item version of the BAT. Based on a sample of 2277 workers, our results supported the factorial validity of a higher-order model represented by 4 first-order factors corresponding to the core dimensions of burnout, namely exhaustion, mental distance, and emotional and cognitive impairment. The measure invariance of the BAT-12 between data collected before and during the COVID-19 pandemic was supported. However, ANCOVA results suggest a higher score on the second-order burnout factor on data collected during the COVID-19 pandemic in comparison with earlier data. In line with the JD-R model, the BAT-12 total score reported a positive association with job demands (i.e., workload, time pressure, and role conflict) and a negative association with job resources (i.e., job autonomy, coworkers’ support) and personal resources (i.e., optimism, social self-efficacy, and task self-efficacy). Additionally, the BAT-12 showed a negative association with work engagement components (i.e., vigor, dedication, and absorption) and positive job attitudes (i.e., job satisfaction, affective commitment). All in all, our results identify the Italian version of the BAT-12 as a brief and reliable tool for measuring burnout among workers.

## 1. Introduction

In the last decades, burnout has become a core concept in occupational health psychology and a matter of global concern owing to its considerable social and economic implications, mainly in terms of productivity loss, sickness absence, and turnover of workers [1]. Academic literature has significantly contributed to a better understanding of this construct, highlighting the adverse consequences of burnout across different domains. Research has consistently provided evidence of poor health among burned-out workers (for a review, [2]).

Unsurprisingly, the World Health Organization recently listed burnout in the 11th revision of the International Classification of Diseases (ICD-11) as a factor that can influence individual health status, although it is not recognized as a genuine medical condition [3].

Christina Maslach [4] is credited with first recognizing the burnout phenomenon. Burnout was not originally identified in the academic context; rather, it emerged as a social problem through interviews focused on how people manage emotional arousal in their daily work. In addition to first labelling this phenomenon and providing the first description of burnout, the pioneering studies conducted since then allowed to enrich theoretical development with empirical assessment. Accordingly, the Maslach Burnout Inventory (MBI) has been universally accepted as a “gold standard” for assessing burnout over the past 40 years. The MBI conceptualizes burnout as a triad consisting of emotional exhaustion, depersonalization, and low personal accomplishment [5]. Specifically, emotional exhaustion refers to an individual’s feeling of being overwhelmed and drained of their energies by their work. Depersonalization entails detachment toward work, colleagues, and clients and perceiving one’s work as meaningless; reduced professional efficacy reflects one’s perceived inadequacy and lack of accomplishment in attaining work purposes.

Despite the abundant research on burnout, mainly based on the MBI, several gaps regarding the measurement and conceptualization of the burnout construct call for an upgrade. For instance, the lack of general consensus on its classification and the absence of formal diagnostic criteria prevented its inclusion in the DSM-5 [6]. Advancements in the assessment tools are vital for adequate screening and, consequently, the implementation of the measures to prevent or counteract burnout occurrence [7]. Moreover, the dimensionality of burnout has been further questioned after broadening the construct beyond the helping professions. The alleged overlapping between cynicism and depersonalization has caused considerable skepticism among scholars who frequently claimed its distinctiveness [8,9]. Reduced professional efficacy has been identified as a consequence of burnout, rather than as a core component, which is empirically unrelated to emotional exhaustion and cynicism [10]. Additionally, the MBI and other traditional measures ignore further burnout symptoms consistently identified, such as lack of concentration and attention [11]. Moreover, calculating and considering each subscale separately, instead of a combined overall burnout score [12], results in questionable practical utility of the instrument for diagnostic purposes and for creating risk thresholds useful for identifying workers who would benefit from preventive interventions.

A recent attempt to overcome these limitations came from Schaufeli, De Witte, and Desart [13] with the development of the Burnout Assessment Tool (BAT). BAT’s underpinning theoretical framework conceptualizes burnout as a syndrome combining four interrelated core components. As per this perspective, *exhaustion* refers to physical and psychological resource depletion. *Mental distance* describes the indifference toward one’s work and disenchantment with its meaning; *emotional impairment* involves overwhelming negative emotions associated with daily tasks; *cognitive impairment* involves signs of impaired cognitive processes, such as focus, attention, and memory. Besides the four dimensions of burnout, BAT covers two secondary symptom subscales: psychological distress (e.g., anxiety symptoms and sleep problems) and psychosomatic disorders (e.g., muscular and gastrointestinal disorders, headaches).

The original version of BAT included 33 items measuring the core components (23 items) and the secondary symptoms (10 items). BAT has been translated and validated across several countries [14,15] and beyond [16,17,18]. Consiglio, Mazzetti, and Schaufeli empirically supported the reliability and factor validity of the Italian version of BAT and the convergent and discriminant validity compared to MBI [19]. According to these findings, burnout could be better explained as a genuine syndrome stemming from a combination of four core components (i.e., exhaustion, mental distance, cognitive impairment, and emotional impairment) belonging to a unique higher-order burnout factor.

The measurement of the four key components of burnout is especially useful as it covers unique expressions of this syndrome. Furthermore, the employment of short psychological scales has been extremely functional in research activities and practice. They save respondents’ time and energy, increasing the compliance rate, thus overcoming momentary and retrospective perceived burden, lower compliance, and lack of caring associated with long measures [20]. The current study explores the validity of the Italian version of BAT-12, consistent with the effort of the BAT consortium [21,22,23].

Therefore, the first hypothesis was developed as follows:

**Hypothesis** **1.** *The factor structure of BAT-12 is best explained as a second-order model with four first-level components (i.e., exhaustion, mental distance, cognitive impairment, and emotional impairment) loading on a higher-order factor (i.e., burnout syndrome).*

Founded on the internal structure of the instrument, further proof of validity entails measurement invariance, a prerequisite for any group comparison based on the Italian BAT-12. Cross-national measurement invariance of BAT across seven national representative samples (i.e., Austria, Belgium, Finland, Germany, Ireland, the Netherlands, and Japan) indicated BAT as a reliable tool to compare burnout levels across countries [15]. Thus far, measurement invariance between burnout levels before and during the COVID-19 pandemic remains to be investigated. Conversely, comprehensive reviews [24,25] on the psychological effects of COVID-19 on the general population have demonstrated severe symptoms, such as increased anxiety, depression, stress, and post-traumatic stress disorder. During the pandemic, employees face exacerbated work-related burdens and cope with aggravated demands across life domains, such as parenting-related exhaustion [26] and increased interpersonal conflicts [27].

Studies reported adverse effects of COVID-19 on employees’ psychological well-being in different work settings and occupations [28,29]. The most severe outcomes of COVID-19 were reported in teaching and healthcare occupations. A systematic review revealed that particularly teachers experience substantial stress, anxiety, and depression [30]. These adverse psychological effects and increased work demands result in severe consequences, such as burnout. Another prominent occupation under critical risk is the healthcare profession. An investigation involving 44 countries revealed that healthcare professionals show a higher increase in stress symptoms than other employees [31]. A global review of the psychological effects of COVID-19 in 35 countries revealed a high prevalence of anxiety (from 22% to 33%) and depression (from 18% to 36%) levels among healthcare professionals [32].

Studies conducted before and during the pandemic reveal alarming results, reporting staggeringly higher levels of burnout among healthcare workers [33]. A large-scale global study conducted among 60 countries during the COVID-19 pandemic revealed that more than half of the healthcare professionals worldwide experienced burnout, which is much higher than the numbers reported by previous studies (before the pandemic) [34]. Based on this empirical evidence, the current study hypothesizes that the Italian BAT-12 presents measurement invariance among data collected before and during the COVID-19 pandemic. However, higher levels of burnout are likely to be expected from data collected during the pandemic.

**Hypothesis** **2.** *The core symptoms of burnout assessed with the Italian version of BAT-12 show measurement invariance between data collected before and during the COVID-19 pandemic.*

To further explore the psychometric properties of the Italian BAT-12, its construct validity was assessed using the Job Demands-Resources (JD-R) model [35] as a conceptual framework. According to the JD-R model, employees’ well-being is determined by (1) *job demands*, as an aspect of one’s job requiring physical and psychological effort; and (2) *job resources*, as “protective factors” enabling employees to meet job demands and encouraging learning and development [36]. Job demands and job resources initiate two distinct processes. Following the health impairment process, persistent exposure to excessive job demands may lead to job burnout when adequate job resources are lacking. Job resources, in contrast, are the main trigger of the motivational process. Thus, they fuel the level of employees’ work engagement and subsequent outcomes, primarily in terms of commitment and superior job performance.

Job burnout is negatively related to several positive job attitudes. Among them, work engagement is defined as a positive, fulfilling, work-related psychological state that combines three interrelated dimensions: vigor, dedication, and absorption [37]. Moreover, burnout is negatively related to affective commitment (i.e., employees’ emotional attachment to the organization), which spurs them on to pursue effective courses of action that support organizational goals [38]. Furthermore, burnout has a negative association with job satisfaction, which can be loosely defined as an individual’s positive affective reaction to the target environment resulting from evaluating the degree to which their needs are satisfied by that environment [39]. Resources can moderate the health impairment process by buffering the effect of job demands on job strain [40]. This effect is not limited to job resources, but also refers to positive self-evaluations concerning the ability to control and impact the work environment (i.e., personal resources).

Earlier research using the JD-R model has shown that BAT scores are positively associated with job demands across several countries (i.e., Japan, the Netherlands, and Belgium) [14,18]. Simultaneously, core symptoms of burnout report a reversed association with job resources (e.g., role clarity), personal resources (e.g., self-efficacy), and positive work-related attitudes (e.g., organizational commitment) [41]. The current study aims to delve deeper into the nomological network of burnout as measured by BAT-12. Accordingly, the following hypothesis was developed:

**Hypothesis** **3.** *Burnout core symptoms, as assessed through the Italian version of BAT-12, show a positive association with job demands (i.e., workload, time pressure, and role conflict) and a negative relationship with job resources (i.e., job autonomy, co-workers’ support) and personal resources (i.e., optimism, social self-efficacy, task self-efficacy) as well as a negative association with positive work-related outcomes (i.e., job satisfaction, affective organizational commitment, and work engagement components).*

## 2. Materials and Methods

### 2.1. Participants and Procedure

The overall sample used in this study comprised 2277 participants working in different organizations that accepted to participate in an organizational well-being survey project. Participants were 57.4% females, 59% were aged between 31 and 50 years, and 50.3% were graduates. Regarding occupational characteristics, 58% had a tenure up to 10 years, 74.6% had a permanent contract, and 55.7% were full-time workers. Regarding the work sector, 41.5% worked in public administration, 26.4% worked in the health and social services and law enforcement, and 14.4% worked in the educational sector. Data collection took about two years: it started before the pandemic in February 2019 lasting one year, then stopped for a few months during the acute phase of the pandemic in Italy (from February to May 2020), and then it started again in June 2020 until February 2021. All in all, 57.5% of the sample questionnaires were collected before the pandemic and 42% during the pandemic. Table 1 provides a full description of respondents’ socio-demographic and occupational characteristics.

Members of the research team contacted different organizations to invite them to participate in an occupational health survey. To maximize data collection, in the case of large organizations, researchers were allowed to adapt the questionnaire by selecting, from a larger list of scales, the ones to include in the survey, except for BAT-12, which was mandatorily included. Therefore, nine slightly different versions of the questionnaire were created. The questionnaire was administered using the online platform Qualtrics (www.qualtrics.com/it; accessed on 1 January 2019). Participants received an e-mail containing an anonymous link allowing them to fill in the online questionnaire. The e-mail included a brief cover letter presenting the following research goals: validating a new burnout questionnaire and exploring its relationships with work-related stress risk and protective factors. Participants’ anonymity and confidentiality were guaranteed, following the guidelines for personal data processing defined by the Italian privacy law (Legislative Decree no. 101 of 10 August 2018). Further, the letter specified that participation was anonymous and voluntary and that participants could withdraw at any time without any requirement to justify their decision.

### 2.2. Measures

#### 2.2.1. Burnout

We measured burnout using the short version of BAT-12 defined according to content and Rasch analysis results [23]. This scale comprised a subset of items taken from the already validated Italian version of BAT-23 [19]. Specifically, three items were used to measure each of the four core burnout symptoms, namely, exhaustion (e.g., “*At work, I feel mentally exhausted*”), mental distance (e.g., “*I struggle to find any enthusiasm for my work*”), cognitive impairment (e.g., “*At work, I have problems staying focused*”), and emotional impairment (e.g., “*At work, I feel unable to control my emotions*”). Participants were asked to answer items indicating the frequency of these symptoms on a 5-point Likert scale ranging from 1 (never) to 5 (always).

#### 2.2.2. Job Demands

Three job demands were measured: time pressure, role conflict, and workload. Time pressure was measured by three items defined by Consiglio et al. [42] (e.g., “*In my job, I constantly feel time pressure*”); role conflict was measured by four items taken from the scale by Bowling and colleagues [43] (e.g., “*In my job, I have to deal with conflicting demands*”). Both scales were rated on a Likert scale ranging from 1 (never) to 5 (always). Workload was measured through five items adapted from work-life areas [44] (e.g., “*My job requires that I work intensively for long periods of time*”), rated on a Likert scale ranging from 1 (completely disagree) to 7 (completely agree).

#### 2.2.3. Job Resources

Two job resources were measured: job autonomy and coworker and supervisor support. Job autonomy was measured through three items from an adapted version of the Morgeson and Humphrey scale [45] rated on a Likert scale ranging from 1 (completely disagree) to 7 (completely agree); this is an example of an item: “*In my job, I decide how to organize activities*.” Support received from colleagues was measured through four items from the coworker’s support scale from the Italian version of HSE’s Management Standard Indicator Tool [46] (e.g., “*Colleagues give me the help and support I need*”) rated on a Likert scale ranging from 1 (never) to 5 (always).

#### 2.2.4. Personal Resources

Three personal resources were measured: optimism, task self-efficacy, and social self-efficacy. Optimism was measured through three items taken from the Italian version of the Psychological Capital Questionnaire [47,48] (e.g., “*When things are uncertain in my line of work, I usually expect them to work out for the best*”), rated on a Likert scale ranging from 1 (never) to 5 (always). Self-efficacy was measured through items taken from an existing work self-efficacy scale [49] preceded by the opening sentence “*At work, I feel I am able to*...” Four items assessed social self-efficacy (e.g., “*I express my opinion during meetings, even when it differs from that of others*”), and four items assessed task self-efficacy (e.g., “*I meet my business deadlines, even when I’m overloaded*”). Both scales were rated on a Likert scale ranging from 1 (cannot do at all) to 7 (highly certain can do).

#### 2.2.5. Positive Job Attitudes

In terms of positive job attitudes, work engagement dimensions, job satisfaction, and affective organizational commitment were measured. Work engagement dimensions were assessed using the Italian version of UWES-9 [50,51], which comprises three items for each of the following scales: vigor (e.g., “*At my work, I burst with energy*”), dedication (e.g., “*I am proud of the work I do*”), and absorption (e.g., *When I am working, I forget everything else around me*). Items were assessed on a 5-point frequency scale ranging from 1 (never) to 5 (always). Job satisfaction was measured through four items. Each item referred to different aspects of the job: activities, relationships with colleagues, relationships with supervisors, and work contests (e.g., “*I am satisfied with my work activity*”). Affective commitment was measured through three items adapted from Mayer and Allen’s scale [52] (e.g., “*I am strongly identified with my organization*”). Affective commitment and satisfaction were rated on a Likert scale ranging from 1 (completely disagree) to 7 (completely agree).

### 2.3. Strategy of Analysis

#### 2.3.1. Confirmatory Factor Analysis

Confirmatory factor analyses were performed on the BAT-12 using the Robust Maximum Likelihood estimator (MLR, robust to non-normality and non-independence of observations) in Mplus 8.1 [53]. The appropriateness of the model fit was established with (1) the Satorra and Bentler [54] scaled chi-square statistic (SB χ^2^), (2) values of CFI and TLI higher than 0.95, (3) RMSEA values lower than 0.06 with associated confidence intervals, and (4) SRMR values lower than 0.08 [55]. In detail, to support the second-order factorial structure of the instrument, four nested models were tested and compared as suggested by Credé and Harms [56]:

- A **single-factor model**, in which all 12 items measuring the four hypothesized core symptoms (i.e., exhaustion, mental distance, emotional impairment, and cognitive impairment) are loaded on a general burnout factor. The test of such a parsimonious model excludes the influences of method bias on observed item covariances [57].
- A **four-correlated factors model**, in which the items loaded on the hypothesized four latent dimensions (i.e., core symptoms) and all of their correlations are freely estimated. This model was tested and compared against the second-order model to assess whether the latter can accurately model the relationships among first-order factors.
- A **second-order model**, in which the four core symptoms are loaded on a higher-order burnout factor that explains the covariations between the first-order factors.
- A **bi-factor model**, in which the items are loaded both onto a general burnout factor and onto the four orthogonal hypothesized core symptoms. This model was tested to exclude whether the correlations among first-order factors are attenuated by differences in how each factor is measured (e.g., content similarities).

The best measurement model was evaluated, for each comparison, through significant differences in the scaled Satorra-Bentler chi-square values (ΔSBχ^2^; *p* < 0.001) [54], by using the DIFFTEST option in Mplus (check for more information: https://www.statmodel.com/chidiff.shtml (accessed on 17 February 2022), and differences in other fit indices.

To corroborate the validity of the hypothesized factorial structure, we tested the measurement invariance of the second-order model between the participants who completed BAT-12 before the COVID-19 pandemic (*n =* 1356) and those who completed it during the pandemic (*n =* 924). According to suggested procedures for multigroup CFA [58], we first tested the goodness of fit of separate baseline models for each group. Then, the model invariance was tested across groups at increasingly stringent levels: (1) configural invariance (i.e., no equality constraints are imposed); (2) metric invariance (i.e., factor loading equality constraints are specified); (3) scalar invariance (i.e., equality constraints on intercepts are specified); and (4) strict invariance (i.e., equality constraints on residuals are imposed). Additionally, following Chen et al.’s indications [59], metric, scalar, and strict invariance were tested for both first- and second-order factors. During each step, the model’s goodness of fit was evaluated, and the significance of measurement invariance was assessed through model differences in the comparative fit index (ΔCFI) with values lower than −0.01, paired with changes in RMSEA of 0.015 and SRMR of 0.030 (for metric invariance) or 0.015 (for scalar and residual invariance) [60]. Finally, we used multigroup CFA to assess whether the second-order factor means differ across the two groups. Once the latent mean of the pre-pandemic group is set to zero, the estimate of the latent mean of the other group, which completed the questionnaire during the pandemic, represents the difference between the factor means in the two groups [60]. The Wald (or z) test was employed to test for significance between the means of the two groups for the latent second-order construct [61].

#### 2.3.2. Analysis of Covariance

To further explore whether there were differences in the means of the BAT-12 total scores between the participants who responded before the COVID-19 pandemic and those who responded during the pandemic, an analysis of covariance (ANCOVA) was conducted by employing the following covariates: (a) gender (i.e., men = 1; women = 2), consistent with a previous meta-analysis that found significant gender differences in terms of burnout [62], and (b) work sectors, which were categorized into two groups: traditional burnout sectors—namely, the human services and educational sectors, which continue to be considered the most exposed to burnout) [63] (i.e., higher burnout risk = 2)—and other private and public sectors (i.e., lower burnout risk = 1).

#### 2.3.3. Internal Consistency

The scale reliability for the overall BAT-12 and its subscale scores and other measures were estimated using Cronbach’s alpha coefficient. As a rule of thumb, values exceeding 0.70 provide evidence of adequate scale reliability [64].

#### 2.3.4. Convergent and Discriminant Validity

To verify the convergent and discriminant validity of the BAT-12 total scores and the specific four core symptoms (i.e., exhaustion, mental distance, cognitive impairment, and emotional impairment), correlations with job resources (i.e., coworkers’ support and job autonomy), personal resources (i.e., task self-efficacy, social self-efficacy, and optimism), job demands (i.e., workload, time pressure, and role conflict), work engagement dimensions, affective commitment, and job satisfaction were investigated on the overall sample by using Pearson’s r coefficient.

## 3. Results

### 3.1. Confirmatory Factor Analysis

Empirical tests (see Table 2) showed that the single-factor model (M1) did not fit the data, while Models 2, 3, and 4 showed a good and similar fit. However, the bi-factor model (M4) fitted the data significantly better than the four-correlated factors model (M2) and the second-order model (M3); moreover, the four-correlated factors model (M2) fitted the data significantly better than the second-order model (M3). These results are consistent with previous validation studies of BAT [19,41].

**Figure 1:**
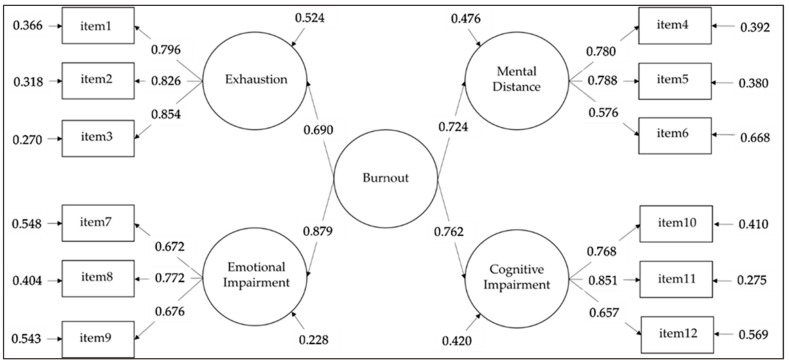
Results of Confirmatory Factor Analysis for the second-order model. Note. The factor-loading matrices of the other tested models and all correlations between the latent variables are available upon request from the first author.

Regarding the second-order model (Figure 1), loadings were significant and above the value of 0.300, ranging from 0.576 to 0.854 for the first-order part of the model and from 0.690 to 0.879 for the second-order part of the model, thus supporting the appropriateness of the factorial validity of the instrument. Correlations among the first-order components of BAT-12 ranged from 0.491 to 0.698.

### 3.2. Measurement Invariance

As shown in Table 3, the hypothesized second-order model fitted the data well for both subgroups of respondents and differenced in terms of the time of administration of BAT-12, that is, pre-COVID-19 pandemic (*n* = 1356) and during the pandemic (*n* = 924). Multigroup analysis supported the measurement invariance of BAT-12 up to the strictest level (i.e., residuals equality holds across groups). Indeed, M1 (configural model), M2 (first-order metric model), M3 (second-order metric model), M4 (first-order scalar model), M5 (second-order scalar model), M6 (first-order strict model) and M7 (second-order strict model) all adequately fit the data. All the differences between subsequent models’ fit indices matched the recommended criteria [60,61]. Furthermore, we found a significant mean difference between the two groups for the second-order factor (0.305, *z* = 39.225, *p* < 0.0001), indicating those who responded to the questionnaire during the COVID-19 pandemic presented higher scores for the second-order burnout factor than the pre-COVID-19 group.

### 3.3. Mean Differences

Table 4 presents the significant differences in the mean values of the BAT-12 total scores among subgroups of respondents, which differs in terms of the time of administration, that is, pre-COVID-19 pandemic (*n* = 1356) and during the pandemic (*n* = 924). The comparison is based on ANCOVA controlling for gender and work sectors that differ in terms of burnout risk. Preliminarily, Levene’s test was not significant (F (1, 2253) = 1.30, *p* = 0.255). As such, the variances for the BAT-12 total scores were homogeneous across the two groups and therefore comparable. We found an overall statistically significant (F = 38.9, *p* < 0.001) difference in the means of the BAT-12 total scores between the pre-COVID-19 group (Mean = 1.82; SE = 0.015) and the during COVID-19 one (Mean = 1.98; SE = 0.018) after adjusting for gender and burnout risk covariates, although such a difference was small in size (η^2^ = 0.018; η^2^p = 0.019).

### 3.4. Reliability and Correlations with Other Dimensions

Cronbach’s alphas of the four core symptoms scales, as well as for the overall BAT-12 scale, were preliminarily calculated on the overall sample and showed good reliability (0.87 for exhaustion, 0.74 for mental distance, 0.75 for emotional impairment, 0.79 for cognitive impairment, and 0.87 for the BAT total score). The reliability of the correlated dimensions was also adequate overall, with alphas ranging from 0.64 (for optimism) to 0.95 (for vigor). As expected, the BAT-12 scales were positively correlated with job demands (see Table 5), namely, workload, time pressure, and role conflict (except for correlations between mental distance and workload and between cognitive impairment and time pressure, which were not significant). Among the four burnout core symptoms, correlations were higher for the association between job demands and exhaustion. Moreover, the BAT-12 scales were negatively correlated with job resources—namely, job autonomy and coworkers’ support (except for the correlation between cognitive impairment and coworkers’ support, which were not significant)—and with personal resources, namely, optimism, task, and social self-efficacy. Among the four core symptoms, correlations were higher for the association between job resources and mental distance. Among personal resources, optimism was more associated with mental distance symptoms, whereas task and social self-efficacy correlated with emotional and cognitive impairment. Finally, the BAT-12 scales were also negatively correlated with work engagement dimensions (i.e., vigor, dedication, and absorption), job satisfaction, and affective commitment, with the only exception being vigor, which was not significantly associated with exhaustion. Nonetheless, an unexpected result entails the non-significant correlation between exhaustion and vigor. This result concurs with previous evidence indicating exhaustion and vigor as unrelated experiences [65]. All significant correlations presented a small to moderate effect size [66], supporting the convergent and discriminant validity of the instrument.

## 4. Discussion

This study aimed to confirm the psychometric properties of the Italian 12-item version of BAT, an instrument grounded in the conceptualization of burnout proposed by Schaufeli et al. [41] and intended to address the shortcomings of leading burnout measures. Consistent with the long Italian version of BAT [19], the results of confirmatory factor analysis provided evidence for a theoretically interpretable 12-item scale consisting of the four core components of BAT (i.e., exhaustion, mental distance, emotional impairment, and cognitive impairment) loading on a unique higher-order factor corresponding to burnout syndrome. These results support *Hypothesis 1*. Accordingly, one of the main novelties of the BAT operationalization of burnout entails the inclusion of reduced cognitive functioning (e.g., concentration and attention) that is empirically identified as a key component of burnout [67] together with emotional impairment, which was also considered a symptom of burnout since early studies [68].

Concerning the questionnaire’s measurement invariance [60], our results showed that constructs were conceptualized similarly before and after the COVID-19 pandemic. On the one hand, before the pandemic, the questionnaire was validated in several countries, and the measurement invariance was tested and showed the equivalence of the strength of the item-factor relations across databases (e.g., countries) [15]. On the other hand, responding to the emerging need for exploring burnout under pandemic conditions [69], both the short and long Ecuadorian versions [21] of BAT have been validated during the pandemic, confirming that the same constructs have been conceptualized and are being interpreted consistently under different circumstances. Similarly, we found agreement regarding how constructs were manifested across the pandemic situation. Furthermore, the structural invariance was supported in such a way that a factorial model among the set of latent variables fits equally well in both samples. Finally, the residual invariance across samples was also supported. Therefore, regarding the comparison of data collected before and after the COVID-19 pandemic, the empirical evidence provided in the current study confirms *Hypothesis 2* and shows the stability of the dimensional structure of the Italian 12-item version of BAT.

Conversely, obtained results revealed a significant mean increase in burnout levels during the COVID-19 pandemic compared to earlier collected data on the second-order factor (i.e., general burnout). This evidence is in line with empirical research that indicates a significant increase in exhaustion, burnout, cynicism, and more negative affective and cognitive responses to change when pre-pandemic and during pandemic psychological states of teachers were compared [70]. Traumatizing experiences faced during the COVID-19 pandemic escalated their burnout and post-traumatic stress levels [71]. Moreover, the adverse effects of the pandemic seem to be continuous and influential in the long run, controlling for the effects of gender and occupational sectors. A longitudinal study conducted on the general working population from various sectors (*n* = 3862) showed that the psychological effects of COVID-19 (such as exhaustion) do not follow a linear pattern; thus, each phase of the pandemic can have its unique characteristics [72].

A further purpose of the current study was to verify the nomological network of burnout, as assessed using the Italian short version of the BAT questionnaire. Consistent with previous studies [22] and with *Hypothesis 3*, the obtained findings show job demands and resources to be positively and negatively, respectively, associated with burnout (BAT-12 total) in the expected direction. Consistent with the JD-R assumptions [35], in the current study, burnout reported a positive association with job demands (i.e., workload, pressure, and role conflict) and a negative association with resources related to one’s job (i.e., job autonomy and colleagues’ support) and personal characteristics (i.e., optimism, social self-efficacy, and task self-efficacy). Therefore, the psychological costs of job demands seem to contribute to a higher BAT-12 total score, whereas job and personal resources are likely protective against burnout. Moreover, the BAT-12 total score was negatively related to positive job attitudes: work engagement dimensions, affective commitment, and job satisfaction.

Overall, it can be concluded that BAT-12 represents a comprehensive factorially valid, reliable, and internally consistent burnout assessment measure also in the Italian context, indicating a psychometrically valid alternative to the already existing instruments. The full version of the Italian BAT-12 is provided in Appendix A (Table A1).

### 4.1. Limitations and Suggestions for Future Research

This study has some limitations that should be considered. First, our convenience sample, even if quite large and heterogeneous, may lead to a sampling bias and is not representative of the Italian working population. In fact, in our sample, some sectors are overrepresented (such as the healthcare and education, and public administration sectors), whereas others are underrepresented (such as industries, retail, and construction sectors). Future studies should use representative samples to balance sector and worker characteristics to provide a more accurate picture of the Italian labor market. Using convenience samples may also increase the possibility that participants who spontaneously decide to adhere to the study are more likely to have low levels of burnout. Moreover, data collected prior to and during the pandemic may differ in terms of contextual and work characteristics due to the work changes caused by COVID-19 widespread. In comparing the mean burnout levels across the two samples, we took into account the effect of gender and work sectors, but not one of the other relevant variables (e.g., remote working). Future studies should control also for those characteristics. However, these contextual differences do not seem to have undermined the construct validity of the BAT across the pandemic.

Another possible weakness is that the BAT scale’s secondary symptoms were not included in this study. We are aware that the secondary symptoms contribute to burnout assessment; however, these scales are not comprised in the short version of BAT-12, and they have already been validated in a previous study [19]. Because BAT was conceived as a diagnostic tool that is able to detect burnout at the individual level, future studies should be targeted to identify clinically validated cut-off values for the Italian working population. However, it is recommended that such studies include workers with overt burnout conditions so as to distinguish low, medium, and high levels of burnout. Moreover, these studies should use the complete version of BAT, comprising the secondary symptoms and clinical interviews for an in-depth analysis of burnout symptoms.

A final weakness of this study is derived from its cross-sectional design. Future studies should explore the test–retest reliability of BAT-12 and longitudinal measurement invariance to test for the stability of the BAT factorial structure over time. This approach would considerably increase the psychometric properties of BAT-12.

### 4.2. Practical Implications

The practical relevance of this study refers to the added value of having a brief, reliable, and sound tool for measuring burnout in the Italian context that can be employed as an alternative to the more widely used MBI. Specifically, the brief measure is particularly suited for organizational surveys and large-scale occupational well-being studies. Thanks to the validation work being carried out in several countries in parallel by the members of the BAT consortium (https://www.burnoutassessmenttool.com/aboutus_eng/#partners; accessed on 1 January 2022), the comparability of burnout data collected across different countries, sectors, and professions will be likely improved. Moreover, the possibility of having an overall burnout score, such as the one provided by BAT, is also particularly relevant for psychosocial risk assessment to assess and compare burnout prevalence across different work settings.

## 5. Conclusions

This study showed the psychometric properties of the Italian version of BAT-12, a new brief tool to assess burnout, using four core dimensions: exhaustion, mental distance, and the new dimensions of emotional and cognitive impairment. The obtained results prove that its factor structure is invariant before and during pandemic working conditions up to the strict level, even if the overall burnout level is higher during the pandemic than before. Additionally, the level of burnout can be summarized in a total burnout score that is related to individual and job characteristics in an expected manner. Hence, the Italian version of BAT-12 represents a sound, short, and practical instrument to assess burnout.

## Figures and Tables

**Table 1:** Sample statistics.

	Total Sample (*n* = 2277)
***Gender***	
Female	57.4%
Male	42.6%
***Age***	
Up to 30 years old	13.9%
From 31 to 50 years old	59.0%
More than 50 years old	27.1%
***Work sector***	
Health, social services, law enforcement	26.4%
Business services	7.7%
Industry	5.1%
Public Administration	41.5%
Educational sector	14.4%
Wholesale or retail trade, repairs	0.6%
Construction	0.4%
Tourism, hospitality, catering	0.6%
Other	3.2%
***Education level***	
Middle School	6.0%
High School	27.0%
University degree	50.3%
Post-graduate degree	16.7%
***Work contract***	
Open-ended contract	74.6%
Fixed-term contract	15.7%
Other	9.7%
***Working hours by contract***	
Full time	55.7%
Part-time	44.3%
***Job tenure***	
Up to 10 years	58.1%
From 11 to 20 years	25.4%
More than 20 years	16.5%
***Time of administration***	
Pre-COVID-19 pandemic	57.5%
During COVID-19 pandemic	42.5%

**Table 2:** Results of confirmatory factor analysis and alternative model comparisons.

	Model Fit						
Model (M)	χ^2^	*df*	Scaling Correction Factor	RMSEA (90% CI)	CFI	TLI	SRMR
**M1: Single-factor model**	2586.476 **	54	1.2813	0.144 (0.139–0.148)	0.705	0.640	0.086
**M2: Four-correlated factors model**	195.829 **	48	1.2342	0.037 (0.031–0.042)	0.983	0.976	0.027
**M3: Second-order model**	218.042 **	50	1.2399	0.038 (0.033–0.044)	0.980	0.974	0.031
**M4: Bi-factor model**	163.79 **	42	1.2244	0.036 (0.030–0.042)	0.986	0.978	0.025
	**Model difference**						
**Model comparison**	**ΔSB χ^2^**	**Δ*df***	**ΔCFI**	**ΔTLI**	**ΔRMSEA**	**ΔSRMR**	
**M2-M1**	1852.94 **	6	0.278	0.336	−0.107	−0.059	
**M3-M2**	20.81 **	2	−0.003	−0.002	0.001	0.004	
**M4-M3**	52.83 **	8	0.006	0.004	−0.002	−0.006	
**M4-M2**	31.58 **	6	0.003	0.002	−0.001	−0.002	

Notes. ** *p* < 0.001; χ^2^ = chi-square statistic; CFI = comparative fit index; TLI = Tuker-Lewis fit index; RMSEA = root mean square error of approximation; SRMR= Standardized Root Mean Square Residual; CI = confidence interval; *df* = degrees of freedom; ΔSB χ^2^ = Satorra-Bentler scaled chi-square difference.

**Table 3:** Results of Tests for Measurement Invariance across Pre-COVID-19 and During COVID-19 groups of respondents.

	Model Fit						
Model (M)	χ^2^	*df*	Scaling Correction Factor	RMSEA (90% CI)	CFI	TLI	SRMR
**Baseline** Pre-COVID-19	157.068 **	50	1.2986	0.040 (0.033–0.047)	0.978	0.970	0.033
**Baseline** During COVID-19	121.031 **	50	1.1681	0.039 (0.030–0.048)	0.981	0.975	0.036
**M1: Configural invariance**	279.923 **	100	1.2304	0.040 (0.034–0.045)	0.979	0.972	0.035
**M2: Metric invariance** (first-order factor loadings invariant)	310.635 **	108	1.2164	0.041 (0.035–0.046)	0.976	0.971	0.040
**M3: Metric invariance** (first- and second-order factor loadings invariant)	317.436 **	111	1.2123	0.040 (0.035–0.047)	0.976	0.971	0.043
**M4: Scalar invariance** (intercepts of measured variables invariant)	348.827 **	119	1.1990	0.041 (0.036–0.046)	0.974	0.971	0.043
**M5: Scalar invariance** (intercepts of measured variables and first-order factors invariant)	411.177 **	122	1.1950	0.046 (0.041–0.050)	0.966	0.964	0.051
**M6: Strict invariance** (residual variances of measured variables)	466.668 **	134	1.2297	0.047 (0.042–0.051)	0.961	0.962	0.061
**M7: Strict invariance** (residual variances of measured variables and first-order factors)	491.095 **	138	1.2327	0.047 (0.043–0.052)	0.959	0.961	0.071
	**Model difference**						
**Model comparison**	**ΔSB χ^2^**	**Δ*df***	**ΔCFI**	**ΔTLI**	**ΔRMSEA**	**ΔSRMR**	
**M2-M1**	32.11 **	8	−0.003	−0.001	0.001	0.005	
**M3-M2**	6.55 (n.s.)	3	0.000	0.000	−0.001	0.003	
**M4-M3**	32.94 **	8	−0.002	0.000	0.001	0.000	
**M5-M4**	70.55 **	3	−0.008	−0.007	0.005	0.008	
**M6-M5**	52.14 **	12	−0.002	−0.002	0.001	0.010	
**M7-M6**	23.64 **	4	−0.002	−0.001	0.000	0.010	

Notes. At each step the prior model served as the baseline against which the subsequent specified model was compared in the sequence of invariance tests, all earlier constraints remained in place; ** *p* < 0.001; χ^2^ = chi-square statistic; CFI = comparative fit index; TLI = Tuker–Lewis fit index; RMSEA = root mean square error of approximation; SRMR = Standardized Root Mean Square Residual; CI = confidence interval; df = degrees of freedom; ΔSB χ^2^ = Satorra-Bentler scaled chi-square difference; n.s. *=* non-significant.

**Table 4:** Results of Analysis of Covariance.

			95% Confidence Interval	
Time of Administration	BAT-12 Adjusted Mean	SE	Lower	Upper
1. Pre-COVID-19	1.82	0.0149	1.80	1.85
2. During COVID-19	1.98	0.0176	1.95	2.02
	**F**	***p***	**η^2^**	**η^2^p**
**Overall model**	38.9	<0 .001		
**Time of administration** (1 = pre-COVID-19; 2 = during COVID-19)	42.7	<0 .001	0.018	0.019
**Occupational sector** (1 = lower risk sector; 2 = higher risk sector)	16.1	< 0.001	0.007	0.007
**Gender** (1 = men; 2 = women)	65.3	< 0.001	0.027	0.028

Notes. The total number of subjects included for gender and burnout risk differed from the total sample size because of missing values. SE = standard error; *p* = statistical significance; F = Fisher statistic; η^2^ = effect size; η^2^p = partial effect size.

**Table 5:** Correlations between the BAT-12 and other dimensions.

Correlated Dimensions (And Related *n* of Respondents)	Mean	*SD*	α	BAT-12	Exhaustion	Mental Distance	Emotional Impairment	Cognitive Impairment
***Workload*** (*n* = 871)	4.10	1.02	0.73	0.267 **	0.413 **	0.058	0.144 **	0.114 **
***Time Pressure*** (*n* = 500)	3.82	1.20	0.78	0.188 **	0.268 **	0.119 **	0.099 *	0.056
***Role Conflict*** (*n* = 386)	2.52	0.89	0.73	0.500 **	0.430 **	0.401 **	0.345 **	0.362 **
***Job Autonomy*** (*n* = 871)	5.11	1.13	0.86	−0.284 **	−0.120 **	−0.336 **	−0.170 **	−0.181 **
***Coworkers’ Support*** (*n* = 485)	3.68	0.86	0.85	−0.163 **	−0.115 *	−0.239 **	−0.108 *	−0.075
***Optimism*** (*n* = 594)	3.75	0.60	0.64	−0.317 **	−0.174 **	−0.344 **	−0.252 **	−0.204 **
***Social Self-efficacy*** (*n* = 862)	5.34	0.98	0.86	−0.317 **	−0.145 **	−0.195 **	−0.300 **	−0.341 **
***Task Self-efficacy*** (*n* = 862)	5.67	0.94	0.89	−0.309 **	−0.156 **	−0.146 **	−0.265 **	−0.406 **
***Job Satisfaction*** (*n* = 871)	5.10	1.24	0.83	−0.477 **	−0.199 **	−0.665 **	−0.239 **	−0.200 **
***Affective Commitment*** (*n* = 871)	5.51	1.10	0.78	−0.346 **	−0.073 *	−0.468 **	−0.186 **	−0.266 **
***Vigor*** ^a^ (*n* = 722)	3.02	0.99	0.95	−0.278 **	−0.034	−0.379 **	−0.126 **	−0.350 **
***Dedication*** ^a^ (*n* = 486)	2.82	0.97	0.93	−0.587 **	−0.503 **	−0.561 **	−0.404 **	−0.495 **
***Absorption*** ^a^ (*n* = 1038)	3.46	1.04	0.90	−0.298 **	−0.097 **	−0.401 **	−0.183 **	−0.327 **

Notes. ^a^ = Dimension of work engagement; * *p* < 0.05; ** *p* < 0.01; SD = standard deviation; α = Cronbach’s alpha coefficient.

## Data Availability

The data that support the findings of this study are available from the corresponding author, G.M., upon reasonable request.

## Appendix A

**Table A1:** Factor loadings and reliabilities of BAT core dimensions.

Items				Factor Loadings			
	*M*	*SD*	*rtot*	Exhaustion	Mental Distance	Emotional Impairment	Cognitive Impairment
Al lavoro mi sento mentalmente esausto/a.	2.63	0.953	0.724	0.796			
Dopo una giornata di lavoro, per me è difficile recuperare le energie.	2.56	0.992	0.751	0.826			
Al lavoro mi sento fisicamente esausto/a	2.30	0.960	0.757	0.854			
Ho difficoltà a provare un qualche entusiasmo per il mio lavoro	2.03	0.973	0.611		0.780		
Provo una forte avversione per il mio lavoro	1.52	0.776	0.621		0.788		
Sono scettico/a rispetto al significato che il mio lavoro ha per gli altri	2.12	1.071	0.484		0.576		
Al lavoro mi sento incapace di controllare le mie emozioni.	1.71	0.763	0.564			0.672	
* Al lavoro mi capita di arrabbiarmi o sentirmi triste senza sapere perché.	1.62	0.792	0.595			0.772	
Al lavoro mi capita di avere delle reazioni esagerate senza volerlo.	1.48	0.650	0.579			0.676	
Al lavoro faccio fatica a mantenere l’attenzione.	1.71	0.727	0.642				0.768
* Quando lavoro ho difficoltà a pensare con lucidità.	1.48	0.606	0.715				0.851
Al lavoro faccio degli errori perché penso ad altro.	1.56	0.605	0.575				0.657
**Cronbach’s α**				**0.866**	**0.735**	**0.748**	**0.794**

Notes. * = These two items in the Italian BAT-12 are taken from the original long version of the questionnaire but differ from those included in the English version of BAT-12. *rtot* = corrected item-total correlation.
